# Supplementary material for: Reduced Tyk2 gene expression in β-cells due to natural mutation determines susceptibility to virus-induced diabetes
Source: Nat Commun. 2015 Apr 7;6:6748. doi: 10.1038/ncomms7748 (PMC4396380; doi:10.1038/ncomms7748)
Supplement: Supplementary Information — Supplementary Figures 1-4 [file ncomms7748-s1.pdf]

Supplementary Data

Supplementary Fig.1

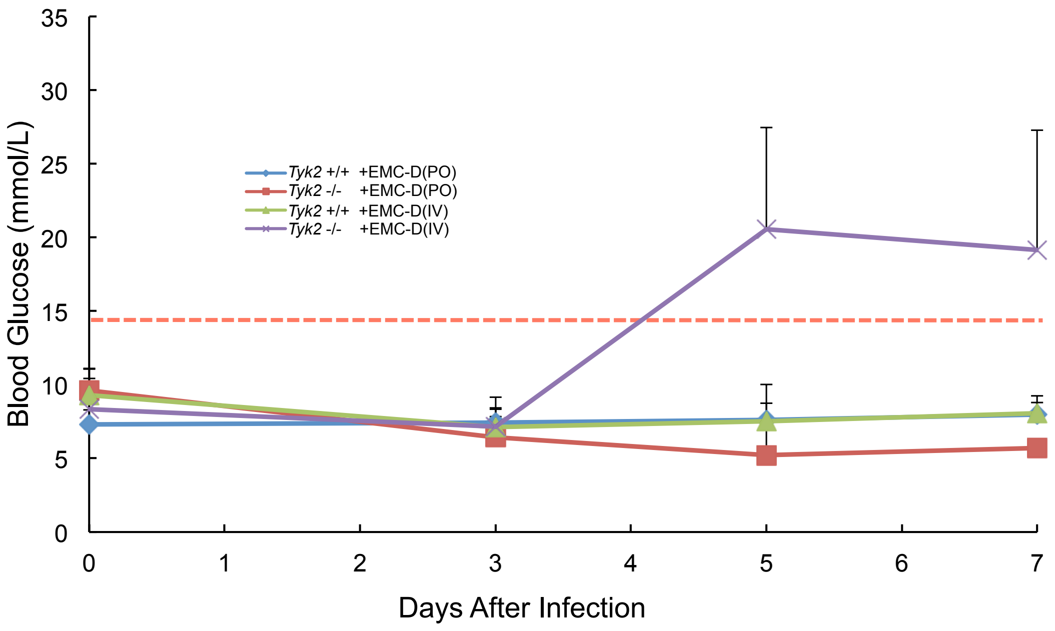


**Supplementary Figure 1. The effect of routes of infection to cause virus-induced diabetes.**

$1x10^3$ p.f.u. of EMC-D virus was given to *Tyk2* WT ($Tyk2^{+/+}$) and KO ($Tyk2^{-/-}$) B6 mice (n=4) by intravenous injection (IV) or oral infection (PO). The blood glucose levels were followed until determination of the development of diabetes. More than 14mmol/L blood glucose level was diagnosed as diabetic (red line). (n=4)

Supplementary Fig.2

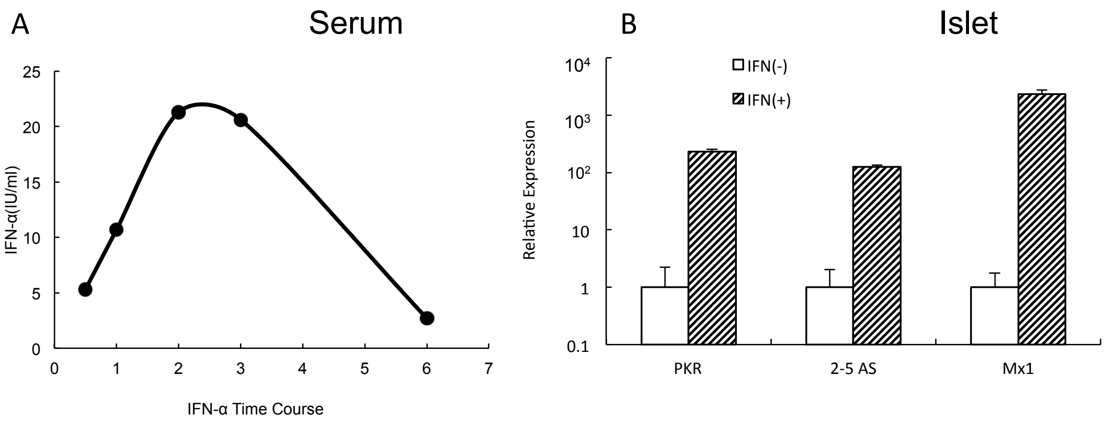


**Supplementary Figure 2. Expressions of IFN stimulated genes (ISGs) in pancereatic islets after IFN-α transfer.**

IFN-α was intraperitoneally injected to WT B6 mice. (A) Assessment of the level of IFN-α in the blood after $1x10^3$ untis of IFN-α transfer. (n=5) (B) Quantification of IFN-stimulated genes (ISGs), such as PKR, 2-5AS and Mx1, using quantitative PCR in islet cells obtained 3 hrs after $1x10^4$ untis of IFN-α transfer. Expression levels of those genes before stimulation was used as a standard. (n=5)

Supplementary Fig.3

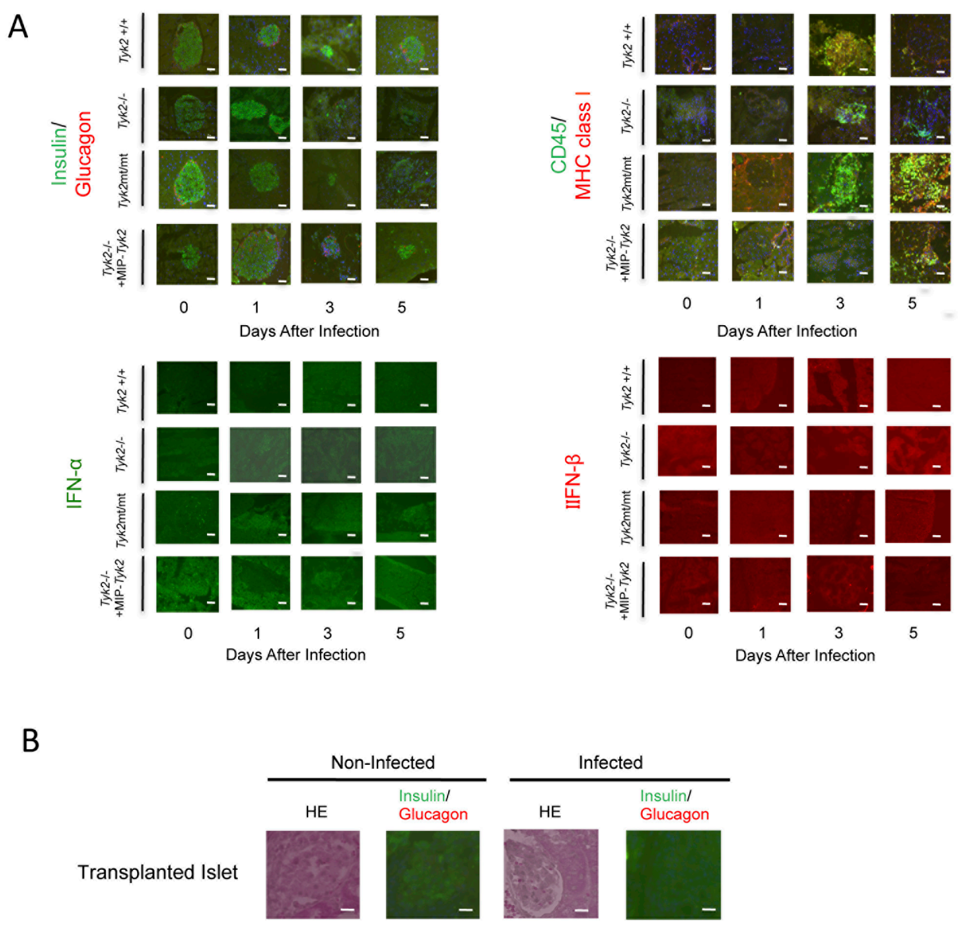


**Supplementary Fig. 3. Immunohistochemical study of islets in mice infected with EMC-D virus.**

(A) Immunofluorescence detection of insulin (green), glucagon red), CD45(green), MHC class I (red), IFN-α (green), and IFN-β (red). Wild type B6, *Tyk2* KO (Tyk2-/-) /B6, *Tyk2* mutated (Tyk2mt/mt) /B6, and *Tyk2* KO (Tyk2-/-) *MIP-Tyk2* Tg/B6 mice were used. All mice were studied after intraperitoneal infection of $1x10^3$ p.f.u. of EMC-D virus. Scale bar, 50μm. (B) Transplanted islets derived from *Tyk2* KO/B6 mice to renal capsule of WT B6 mice were stained with insulin (green) and glucagon (red), 5 days after intraperitoneal infection with $1x10^3$ p.f.u. of EMC-D virus. Scale bar, 50μm.

Supplementary Fig.4

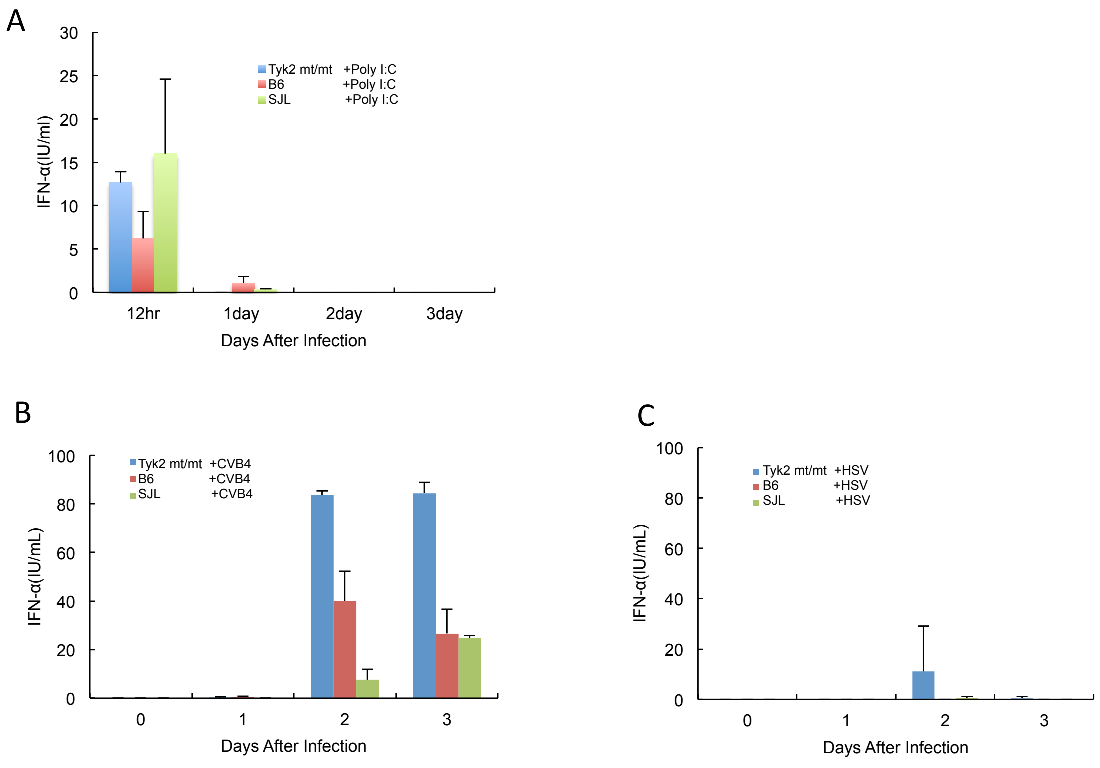


**Supplementary Figure 4. Induction of IFN-α in various strains of mice as B6, SJL, and *Tyk2* mutated B6 mice.**

(A) 250 ug of poly I:C was given intraperitoneally. (B) $1x10^3$ p.f.u. of coxsackie B4 was intraperitoneally injected. (n=3) (C) $1x10^3$ p.f.u. of HSV was intraperitoneally administered to the mice. Levels of IFN-α were measured by ELISA. (n=3)
